# Agraphia: Presenting Feature of Syndrome of Transient Headache and Neurological Deficits With Cerebrospinal Fluid Lymphocytosis (HaNDL)

**DOI:** 10.7759/cureus.13178

**Published:** 2021-02-06

**Authors:** Jaime Leonardo I Salazar-Orellana, Gala Prado-Miranda, Amelia Maldonado-Ortiz

**Affiliations:** 1 Department of Neurology and Psychiatry, Instituto Nacional de Ciencias Médicas y Nutrición Salvador Zubirán, Mexico City, MEX; 2 Department of Neurology, Hospital General de México Dr. Eduardo Liceaga, Mexico City, MEX

**Keywords:** handl, lymphocytic pleocytosis, spect, headache disorders

## Abstract

We report a case of a 48-year-old man with a history of episodes of severe headache, accompanied by motor aphasia and agraphia, with complete recovery between episodes. The neurological examination revealed no abnormality. A lumbar puncture was performed and showed lymphocytic pleocytosis. Cerebrospinal fluid analysis ruled out viral, bacterial, mycobacterial, fungal, treponemal, and NMDA receptor antibodies. Brain magnetic resonance imaging and electroencephalogram revealed no abnormalities. A focal frontotemporal area of hypoperfusion was detected in brain single-photon emission tomography. A diagnosis of syndrome of transient headache and neurological deficits with cerebrospinal fluid lymphocytosis (HaNDL) was made to the patient and then treated with ibuprofen 400 mg three times per day with excellent response. He remained asymptomatic and free of any relapse during six months of following. We presented a typical case of HaNDL that manifests with agraphia, a transient focal neurological deficit non previously reported. Showing that the clinical picture could probably be any sign and symptom related to focal cortical alteration due to cortical transient hypoperfusion.

## Introduction

The syndrome of transient headache and neurological deficits with cerebrospinal fluid lymphocytosis (HaNDL) was first described in 1981 in seven patients with migraine-like episodes associated with abnormalities in the cerebrospinal fluid (CSF), predominantly lymphocytic pleocytosis [1]. HaNDL, also known as pseudomigraine with temporary neurological symptoms and lymphocytic pleocytosis, is a rare disease. The clinical presentation may resemble some serious neurological disorders, including stroke and viral encephalitis.

HaNDL often presents in patients during the second to fourth decades of life, with a viral-like illness three weeks earlier that can precede the onset of the syndrome in 25% of cases [2]. The patient presents with a severe headache and focal neurological deficits that usually last from five minutes to three days, most commonly presenting with motor aphasia and sensory symptoms [2,3]. HaNDL is a diagnosis of exclusion. Diagnosis is made by The International Classification of Headache Disorders, 3rd edition criteria which includes: (1) episodes of migraine-like headache; (2) accompanied or shortly preceded by the onset of at least one transient neurological deficits lasting >4 hours (hemiparaesthesia, dysphasia, hemiparesis), and cerebrospinal fluid (CSF) lymphocytic pleocytosis (>15 white cells per ml), with negative aetiological studies; (3) headache and transient neurological deficits have developed or significantly worsened in temporal relation to the onset or worsening of the CSF lymphocytic pleocytosis, or led to its discovery, and/or headache and transient neurological deficits have significantly improved in parallel with the improvement in the CSF lymphocytic pleocytosis; (4) not better accounted for by another diagnosis [4].

Herein, we describe a case of HaNDL syndrome who presented with agraphia, a clinical feature not previously described in the literature, that expands the clinical constellation of neurological focalization that could be seen in HaNDL.

## Case presentation

**Figure 1:**
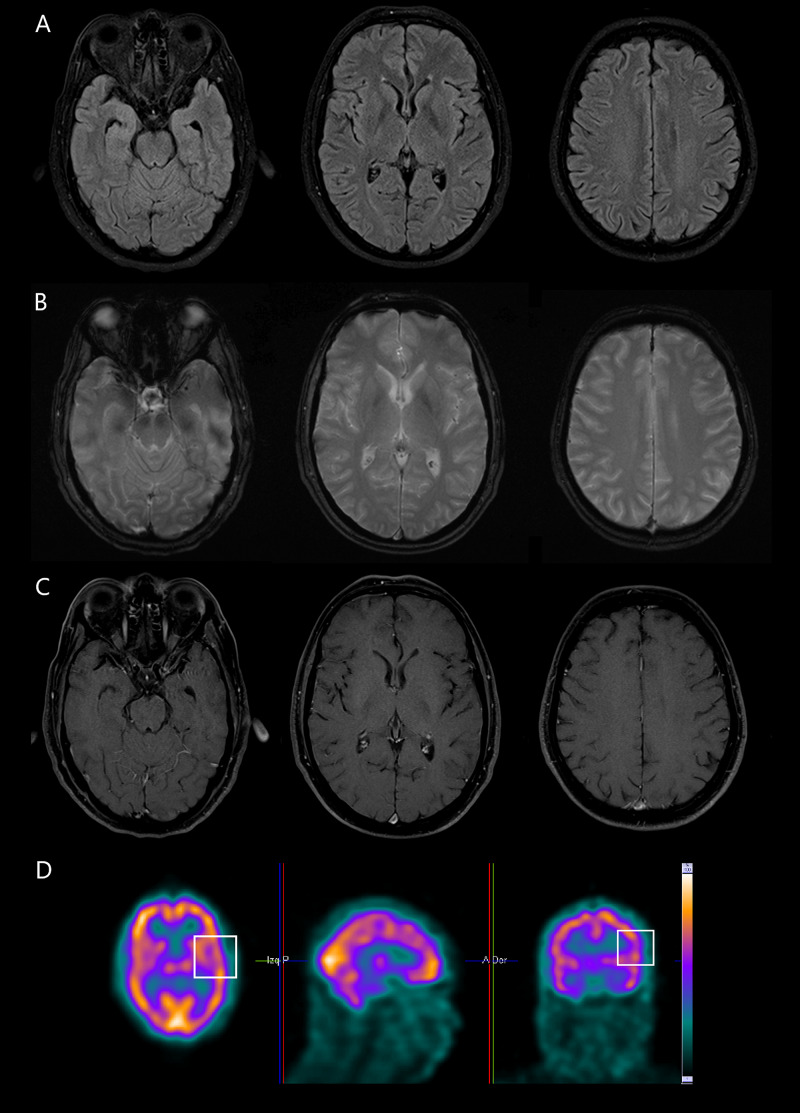
Brain MRI and SPECT. Brain MRI showing neither gray nor white matter lesions in T2-weighted-fluid-attenuated inversion recovery (panel A), lack of hemorrhages or microbleeds in susceptibility-weighted Imaging (panel B), and no cortical or meningeal enhancement in the contrast-enhanced t1-weighted image (panel C). Single-photon emission computed tomography shows a slight decrease in cerebral blood flow in the left frontotemporal cortex (white squares) (panel D). SPECT: single-photon emission computed tomography.

A 48-year-old man, without any past medical history, had an auto limited flu-like syndrome that lasted five days. Two weeks later, he presented to the emergency department with an episode of severe headache, accompanied by nausea, followed 30 minutes later with motor aphasia and agraphia, unable to perform handwrite, and writing text messages on his cell phone. The symptoms resolved 4.5 hours later, returning to normality. Three days later, he had a new episode of similar features, but this time lasting eight hours and then recovering completely. The neurological examination revealed no abnormality. A lumbar puncture and CSF study revealed lymphocytic pleocytosis (112 cells/mm3; 95% lymphocytes), a protein level of 196 mg/dL, a glucose level of 68 mg/dL, and no erythrocytes. CSF microbiological analysis including Gram stain, India ink stain, Xpert MTB/RIF assay, viral polymerase chain reaction for herpes simplex virus 1 & 2, varicella-zoster virus, Epstein-Barr virus, cytomegalovirus, enterovirus, and human herpes virus-6 was all negative; testing for syphilis and Anti-NDMA-receptor antibodies in CSF were also negative. Serological tests were negative for treponema pallidum and human immunodeficiency virus (HIV). His initial blood workup, including a complete blood cell count, renal and liver function, electrolytes, coagulation screen, and C-reactive protein, were all within normal limits. A magnetic resonance imaging (MRI) of the brain was unremarkable. Under suspicion of viral encephalitis, we started treatment with intravenous acyclovir. Three days after admission, the patient experienced a new episode of severe headache, accompanied by agraphia and motor aphasia that last four hours. A second brain MRI was unremarkable (Figure 1A-C).

The patient remained asymptomatic, and a lumbar puncture repeated 10 days later after admission revealed pleocytosis of a lower magnitude (27 cells/mm^3^; 100% lymphocytes), a protein level of 118 mg/dL and glucose level of 64 mg/dL, bacterial, mycobacterial, fungal and viral assessment was again negative. Electroencephalogram showed background rhythm with a frequency of 9.0 to 9.5 Hz and an average amplitude of 25 to 50 mV, and no abnormal activity. HaNDL diagnosis was suspected. A single-photon emission computed tomography (SPECT) looking for focal areas of hypoperfusion was performed on the patient, revealing decreased blood flow in the left frontal cortex (Figure 1). The patient was diagnosed with HaNDL; thus, acyclovir was withdrawn. Treatment with a short course of ibuprofen 400 mg three times per day provided an excellent response, and the patient was discharged twelve days after hospital admission. He remained asymptomatic and free of any relapse during six months of following.

## Discussion

We believed that this is a case of HaNDL because it fulfills the diagnostic criteria. First, the patient had a flu-like syndrome two weeks earlier to his presentation. Second, he presented three severe headaches episodes, accompanied by neurological focalization, specifically agraphia, and motor aphasia, that lasted >4 hours. Third, in the CSF analysis, lymphocytic pleocytosis was discovered, and an extensive aetiological workup revealed no infectious or inflammatory source, including viral, bacterial, fungal, treponemal, and NMDA-receptor antibodies. Also, a systemic cause was discharged (HIV and syphilis). Fourth, the headache and the lymphocytic pleocytosis improved in parallel.

The etiopathogenesis of HaNDL is not fully understood, and various hypothesis has been proposed. A viral etiology has been suggested, but to date, no clear link could be identified. In fact, in nearly all HaNDL cases reported, no virus was founded to support this hypothesis [5]. Due to the possibility that some viral infections can cause temporary neurological deficits and CSF lymphocytic pleocytosis, a complete workup looking for infectious, especially viral etiologies is important in possible HaNDL cases to fulfill the diagnostic criteria. Also, migrainous pathophysiology has been suggested; some patients have a prior history of migraine. SPECT and perfusion imaging with computed tomography (CT) can reveal hypoperfusion without vessel occlusion, suggesting a phenomenon of cortical spreading depression similar to that of migraine with aura [6]. A unified hypothesis suggests an inflammatory mechanism induces leptomeningeal vasculitis, leading to headaches and neurological deficits due to a spreading depression-like mechanism [7].

In a report of 50 patients with HaNDL, 80% (40 patients) had transient neurological deficits restricted to one hemisphere, most of them in the left hemisphere (74%), with a duration of 5 minutes to three days. The most common deficit was sensitive in 70%, followed by aphasia in 66% (motor aphasia in 36%, global aphasia in 30%, and sensitive aphasia in 6%), hemiparesis in 42%, visual symptoms in 18% (homonymous hemianopsia 8%, bilateral blurring of vision 4% and photopsia in 6%). The most common combination was motor aphasia plus sensory and motor right hemibody symptoms [2].

Curiously, the transient focal neurological deficits in our patient include agraphia, a feature not previously described in HaNDL patients, expanding the list of clinical deficits already known that includes motor and sensory aphasia, sensory symptoms, weakness, visual symptoms [2], cranial neuropathy [8], papilledema and acute elevation of intracranial pressure [7], disorientation and inattention [9].

The electroencephalogram showed no abnormal activity, but temporal and frontal slowing and epileptic-like changes can be found [10]. In MRI, typically, there's a lack of abnormal findings, in concordance with patient MRI; but, focal leptomeningeal enhancement, focal areas of hypoperfusion, and decreased prominence of the venous vasculature in susceptibility-weighted Imaging (SWI) can be found [11]. A decrease in cerebral blood flow can be found in SPECT [12] that correlates in 90% of cases with the topography of clinical manifestations [10], as was seen in this patient in whom a slight decrease was found in the left frontotemporal cortex, including the cortical area related to writing (Exner's area), correlating with the clinical finding of agraphia exhibited by the patient.

When a transcranial Doppler is performed in a patient with HaNDL during and after episodes of transient neurological deficit, an asymmetry in the blood flow of the middle cerebral artery can be found, in association with changes in pulsatility, suggesting fluctuation in arteriolar tone and demonstrating a focal vasomotor disturbance as a possible explanation to the transient neurological deficit [13].

Lastly, there is no specific treatment for HaNDL, symptomatic management of headache with analgesics are enough. Given the self-limited nature of HaNDL, no other therapeutic interventions are necessary.

## Conclusions

Here we presented a typical case of HaNDL presenting with transient episodes of agraphia, a transient focal neurological deficit non previously reported. Showing that the clinical picture could probably be any sign and symptom related to focal cortical transient alteration due to hypoperfusion, as was evidenced in SPECT. As HaNDL can mimic other neurological disorders, it is important to consider the possibility of the diagnosis once other entities like stroke and encephalitis have been correctly ruled out.
